# Crocin Improves Cognitive Behavior in Rats with Alzheimer's Disease by Regulating Endoplasmic Reticulum Stress and Apoptosis

**DOI:** 10.1155/2019/9454913

**Published:** 2019-08-26

**Authors:** Ling Lin, Guoliang Liu, Lina Yang

**Affiliations:** ^1^Department of Physiology, Henan Medical College, Zhengzhou 450003, China; ^2^Department of Preventive Medicine, Henan Medical College, Zhengzhou 450003, China

## Abstract

**Aim:**

To investigate the effect of crocin on the learning and memory acquisition of AD rats and its underlying mechanisms.

**Methods:**

A total of 48 healthy male SD rats were randomly divided into control group, AD model group, resveratrol group, and crocin group, with 12 rats per group. AD model was established by injecting A*β*_25–35_ to the lateral ventricle of rats, and thereafter the rats were administrated with resveratrol (40 mg/kg), crocin (40 mg/kg), or PBS daily for 14 days. Y-maze test and sucrose preference test were used to detect the learning and memory acquisition of rats. Neuronal apoptosis was detected by TUNEL staining and Western blot for apoptosis-related proteins Bax, Bcl-2, and Caspase-3. Immunofluorescence staining and Western blot tests were used to detect the expression of glucose regulated protein 78 (GRP78) and C/EBP homologous protein (CHOP) in hippocampal CA1 region (Hippo) and prefrontal cortical neurons (PFC).

**Results:**

The learning and memory abilities of AD rats were significantly decreased, which was significantly rescued by resveratrol and crocin. The apoptotic cell number of Hippo and PFC neurons in AD model group was significantly higher than that in control group (*P*<0.01), while resveratrol and crocin significantly decreased the apoptotic cell number in AD group (*P*<0.01). Compared with the control group, the expression of Bcl2 in PFC and hippo of AD model group was significantly decreased (*P*<0.01), while those of Bax, Caspase3, GRP78, and CHOP were significantly increased (*P*<0.01). Resveratrol and crocin could significantly reverse the expression of these proteins in AD rats (*P*<0.05).

**Conclusion:**

Crocin can improve the learning and memory ability of AD rats possibly by reducing endoplasmic reticulum stress and neuronal apoptosis.

## 1. Introduction

Alzheimer's disease (AD), commonly known as senile dementia, is a common neurodegenerative disease characterized by progressive memory loss [1]. The incidence of AD in elderly population is increasing year by year and is expected to reach 50 million in 2018 [2], which seriously affects the life quality of patients and their families [3, 4]. The main pathological changes of AD are the presence of a large number of senile plaques, amyloid *β* protein deposition, and neurofibrillary tangles in the brain [5]. However, the pathogenesis of AD is not fully understood until now. Moreover, there is no reliable treatment in clinical practice.

Endoplasmic reticulum is an organelle for protein synthesis, translation, modification, and secretion in eukaryotic cells [6]. The accumulation of misfolded proteins in the lumen of the endoplasmic reticulum results in dysfunction of the endoplasmic reticulum, inducing endoplasmic reticulum stress (ERS) [6]. A large number of studies have shown that ERS is closely related to the occurrence of AD [7–9].

The molecular chaperone glucose regulated protein 78 (GRP78) is a specific marker of ERS [10]. When a large number of abnormal proteins are present in cells and bind to GRP78, GRP78 is dissociated from the ERS components (including PKR-like ER kinase (PERK), inositol requiring enzyme 1 (IRE1), and activating transcription factor 6 (ATF6)) to activate these components and finally activate ERS and promote apoptosis [11]. ERS causes cell apoptosis mainly by increasing the transcription level of C/EBP homologous protein (CHOP), in which CHOP is an important signaling molecule to convert survival to apoptosis [12].

Crocin is a non-tetraquinone pigment extracted from* Crocus sativus L.*, and our previous studies showed that crocin can improve the learning and memory acquisition of AD rats [13, 14]. Research has shown that the active ingredient of crocus sativus, trans-crocetin, can contribute to the degradation of A*β*_42_ by mononuclear phagocytes in AD patients; this may be related to the increased expression of cathepsin (CatB) in mononuclear phagocytes of AD patients by trans-crocetin [15]. We also reported that the higher dose (40 mg/kg and 80 mg/kg) of crocin performed significantly better than lower dose (20 mg/kg), but no difference was found between 40 mg/kg and 80 mg/kg crocin. However, whether the improvement effect of crocin on AD rats depends on neuronal apoptosis mediated by endoplasmic reticulum stress is still unknown. Therefore, we determined to study the effect and working mechanism of crocin (40 mg/kg) in AD. Therefore, we determined to study the effect and working mechanism of crocin (40mg/kg) in AD.

In this study, the rat AD model was induced by injection of A*β*_25–35_ in the lateral ventricle, and the treatment effect of crocin on learning and memory was studied. We also investigated the expression of ERS-related proteins, GRP78, and CHOP, in hippocampal CA1 region (Hippo) and prefrontal cortical neurons (PFC), as well as the apoptosis of brain neural cells to investigate the working mechanism of crocin. Our study thus will provide basis for the clinical treatment of AD.

## 2. Material and Methods

### 2.1. Animals

A total of 48 male Wistar rats (180-220 g of weight) were obtained from Hunan SJA Laboratory Animal Co., Ltd (Permit Number: SCXK-Xiang 2011-0003). The animals were housed under special pathogen-free conditions with 12 hr:12 hr light/dark cycle with water and food* ad libitum*. The Institutional Animal Care and Use Committee of Henan Medical University approved all of the experiments.

Animals were randomly divided into four groups with 12 rats per group, including control group, AD model group, resveratrol (40 mg/kg) group, and crocin (40 mg/kg) group.

### 2.2. Alzheimer's Disease Model

A total of 0.1 mg A*β*_25–35_ (Sigma-Aldrich, St. Louis, Missouri) was dissolved in 50 *μ*L DMSO and then diluted by 0.01 M PBS to the concentration of 10 mg/ml as a stocking solution. A*β*_25–35_ was “aged” by incubation at 37°C for 1 week and stored at −20°C for use. The AD model was established by intracerebroventricular administration of A*β*_25–35_. All the rats were anesthetized with 10% chloral hydrate (300 mg/kg) and fixed on the brain stereotaxic apparatus (Anhui Zhenghua Technology co., LTD). The skin of head was cut and dura mater was exposed. The injection volume of condensate A*β*_25–35_ (5 *μ*L) was gradually injected within 10 min with a microsyringe (Chengdu Taimeng Technology co., LTD) to the right ventricle according to the rat brain in stereotaxic coordinates (0.8 mm behind the bregma and 1.8 mm beside the midline; vertically insert the needle into 3.5 mm below the dura mater); the needle was then kept for 30 min and slowly withdrawn. The control group of rats was administrated using the same method with saline. After the intracerebroventricular administration, all the rats were administrated with penicillin before suture.

The resveratrol group and crocin group of rats were intraperitoneal injection with 40 mg/kg resveratrol or 40 mg/kg crocin daily for 14 consecutive days from the next day of intracerebroventricular administration of *αβ*_25–35_. The resveratrol (94238-00-3) and crocin (29700-22-9) were purchased from Nanjing Jingzhu Biological Technology Co., Ltd. The control group and AD group of rats were administrated with equal volume (10 *μ*L) of PBS.

### 2.3. The Y-Maze Test

At the end of resveratrol and crocin administration, responses of mice to behavior tasks were performed. The Y-maze task included a first phase (learning test) in which the correct reaction is entering the safe zone from the start zone when rats received an electric shock. The learning acquisition is the times of shock to achieve 9 correct reactions in a consecutive of 10 tests, and rats that tried more than 100 times were excluded. The animals were then subjected to the next phase (memory retrieval) test. After 24 hr of resting, rats were tested as in the learning test phase to calcite their memory retrieval acquisition. The correct reaction rate was calculated by dividing the times of correct reactions by the total times of test.

### 2.4. Sucrose Preference Test

The sucrose preference test was performed in two stages. At the training stage, the rats in each group were housed separately and were offered 200 mL 1% sucrose water and regular drinking water at the end of resveratrol and crocin injection. After 24 hr of fasting, the rats were provided with 200 mL 1% sucrose water and regular drinking water for the test stage. The position of the two bottles was exchanged after 24 hr. The bottles were weighed prior to being given to the rats and at the conclusion of the test. The percentage of sucrose preference = sucrose consumption / (sucrose water + regular water) x 100%.

### 2.5. TUNEL Assay

Six rats randomly from each group were perfused with 4% paraformaldehyde, and the brain was taken. The hippocampal CA1 area and the prefrontal cortex were separated under microscopy and placed into 4% paraformaldehyde overnight. After being dehydrated by 30% sucrose and sliced by frozen slicer (Leica, Germany) into 15 *μ*m sections, the sections were immersed in 10% goat serum (Beyotime Biotechnology, China) for blocking. Then the sections were permeabilized with 0.3% Triton X-100 for 2 hr, followed by 10% BSA blocking for 1 hr. After washing with 0.01M PBS for 3 times, the sections were incubated for 60 min with TUNEL reaction mixture in the TUNEL assay kit (Beyotime Biotechnology, China) according to the manufacturer's instruction. The FITC-labeled TUNEL-positive cells (green) were calculated under a fluorescent microscopy (OLYMPUS, Japan) by using 488-nm excitation and 530-nm emission wavelengths.

### 2.6. Immunofluorescent Staining

The brain sections from the same 6 rats were blocked by goat serum, treated with Triton X-100, and blocked by 10% BSA, as described above. Then sections were incubated with primary antibody that was targeted against CHOP (mouse polyclonal, 1:500; Abcam, UK), GRP78 (rabbit polyclonal, 1:500; Abcam,UK) at 4°C overnight. After washing with 0.01 M PBS, FITC labeled goat anti-rabbit and Cy3 labeled goat anti-mouse secondary antibodies were added and incubated in dark for 2 hr at room temperature. After washing with 0.01M PBS for 3 times, nuclei staining was performed with DAPI (Beyotime Biotechnology, China), and the fluorescent positive cells were observed under a fluorescent microscopy (OLYMPUS, Japan). The average number of fluorescent positive cells and the integrated optical density (IA) in prefrontal cortex and CA1 region of hippocampus were calculated by image pro plus 6.0 (Media Cybernetics, USA).

### 2.7. Western Blotting

The brains of the other half of rats were taken after anesthesia. Cortical tissue was removed, and the hippocampus was separated out and stored in liquid nitrogen. The frozen hippocampus samples were ground and lysed in RIPA lysis buffer and Protein Quantitative Kit (Beyotime Biotechnology, China). A total of 50 *μ*g protein was separated by SDS-PAGE and transferred to PVDF membranes. The membranes were blocked for 1 hr at room temperature with skimmed milk and then probed for overnight at 4°C using a primary antibody that was targeted against CHOP (mouse polyclonal, 1:500; Abcam, UK), GRP78 (rabbit polyclonal, 1:500; AbcamUK), Bax (rabbit polyclonal, 1:500; Abcam,UK), Bcl-2 (rabbit polyclonal, 1:500; Abcam,UK), Caspase-3 (rabbit polyclonal, 1:500; Abcam,UK), or GAPDH (rabbit polyclonal, 1:1000; Abcam,UK). The membranes were then incubated with horseradish peroxidase-conjugated anti-rabbit IgG (1:2000; Beyotime Biotechnology, China) or anti-mouse IgG (1:2000; Beyotime Biotechnology, China) for 2 hr at room temperature. The bands were visualized using a chemiluminescence-based detection kit (Beyotime Biotechnology, China) and the OD value was quantified using Image Proplus 6.0 analysis system (Media Cybernetics, USA).

### 2.8. Statistical Analysis

Data was analyzed using SPSS 24.0 statistical software (SPSS Statistics/IBM Corp, Chicago, IL, USA). Differences among groups were analyzed using one-Way ANOVA post hoc test, and post-ANOVA multiple comparison tests were performed by SNK-q. All results were presented as mean ± standard deviation (Mean ± SD).* P*-values <0.05 were considered statistically significant.

## 3. Results

### 3.1. Effects of Crocin on Learning and Memory Abilities of AD Rats

**Figure 1:**
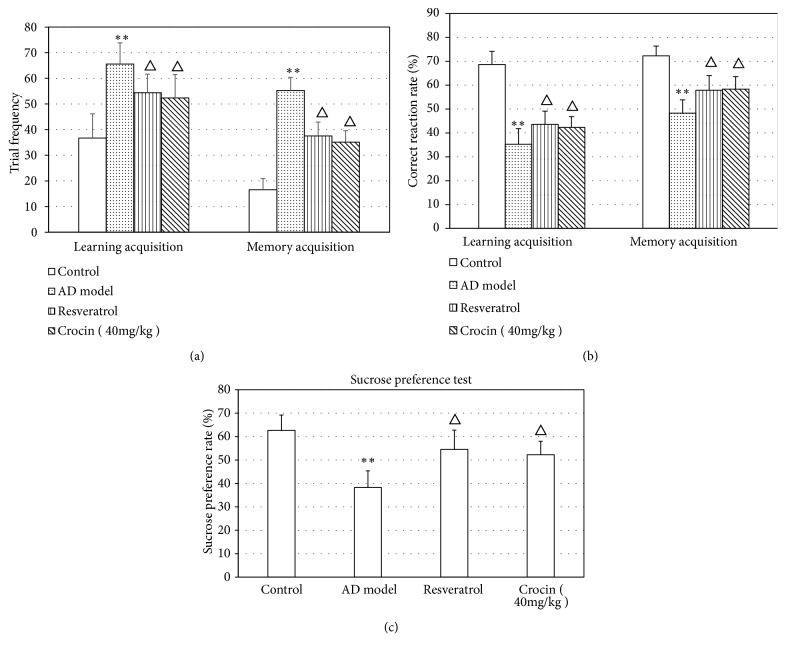
Comparison of learning and memory ability in each group of rats. At the end of resveratrol and crocin administration, responses of rat to behavior tasks were performed. The trial frequency (a) and correct reaction rates (b) in both learning and memory stages were analyzed using Y-maze test. (c) Sucrose preference of rats was determined by sucrose preference test. Data was shown as Mean±SD. *∗*P<0.05 vs control group; ^△^P<0.05 vs AD model group.

We first analyzed whether crocin could improve the learning and memory abilities of AD rats. We showed that in both learning and memory stages the attempting times of AD rats in Y-maze test were significantly increased comparing with the control rats (P<0.01; Figure 1(a)), and the correct reaction rate was significantly decreased (P<0.01; Figure 1(b)). However, rats in crocin group showed significantly less attempt times and high correct reaction rate (P<0.01; Figures 1(a) and 1(b)). In sucrose preference test we also found significantly less sucrose preference in AD rats when compared with control rats (P<0.01), which was rescued by crocin administration (P<0.01; Figure 1(c)). Moreover, the effect of crocin and resveratrol on AD rats was similar. There results indicate that crocin could improve the learning and memory abilities of AD rats.

### 3.2. Effects of Crocin on Apoptosis of Hippocampal CA1 Region and Prefrontal Cortical Neurons

**Figure 2:**
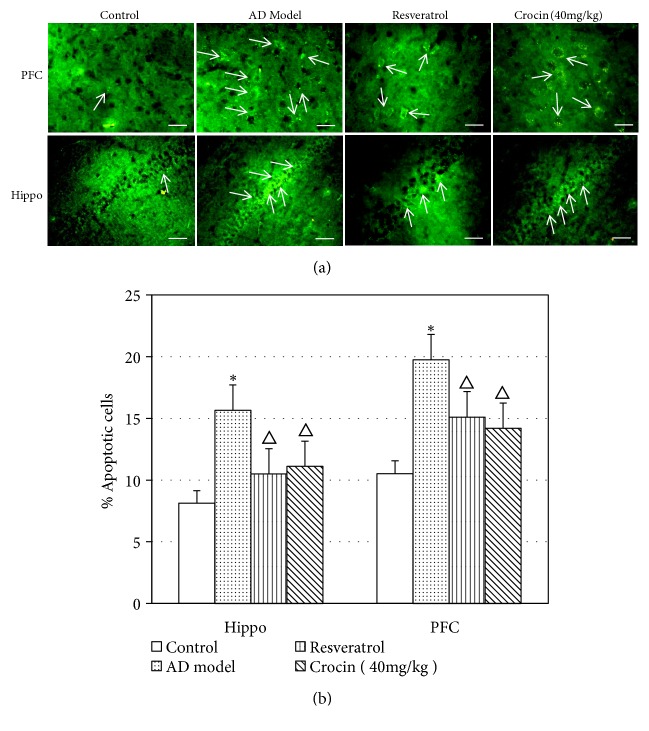
TUNEL staining to detect apoptosis of hippocampal CA1 region (Hippo) and prefrontal cortical (PFC) neurons in rats of each group. (a) Apoptotic cells in Hippo and PFC detected by TUNEL staining. Scale bar = 20 *μ*m. (b) Comparison of apoptotic neurons number in Hippo and PFC in each group of rats (n=6). Data was shown as Mean±SD. *∗*P<0.05 vs control group; ^△^P<0.05 vs AD model group.

TUNEL assay was then performed to investigate the effect of crocin on neural cell apoptosis. As shown in Figure 2(a), the green florescent apoptotic cells could be clearly observed in hippocampal CA1 region (Hippo) and prefrontal cortical neurons (PFC) of rat brain. Tissues from AD rats had significantly higher number of apoptotic cells compared with control rats. In contrast, both crocin and resveratrol significantly decreased the number of apoptotic cells in AD rats (Figure 2(b)).

**Figure 3:**
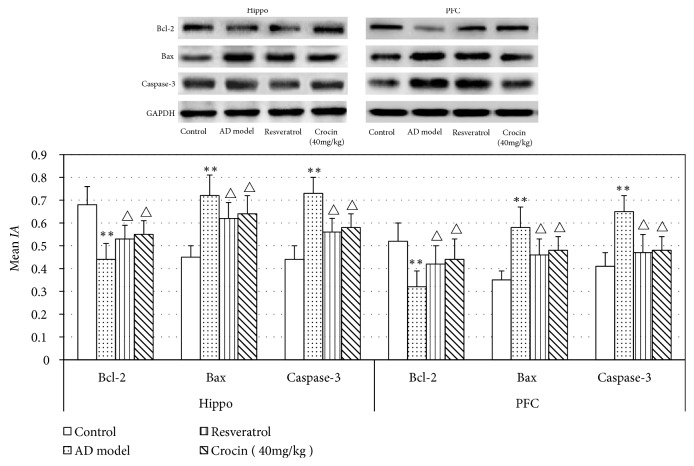
Western blot analysis of Bax, Bcl-2, and Caspase-3 protein expression in Hippo and PFC in each group of rats (n=6). The relative expression of protein was analyzed by comparing with the expression of GAPDH. Data was shown as Mean±SD. *∗*P<0.01 vs control group; ^△^P<0.05 vs AD model group.

To further confirm the effect of Crocin on cell apoptosis in hippocampal CA1 tissues and PFC, Western blot was performed to analyze the expression of apoptotic related proteins in these tissues. We observed significantly decreased expression of Bcl-2 but higher expression of Bax and Caspase-3 in AD rats comparing with that in the control group (*P* <0.01; Figure 3). As expected, crocin and resveratrol significantly attenuated the expression changes of apoptotic related proteins (*P* <0.05; Figure 3). There results indicate that crocin can reduce the apoptosis of neurons in Hippo and PFC of AD rats, increase the expression of Bcl-2, and reduce the expression of Bax and Caspase-3.

### 3.3. Effects of Crocin on ERS-Related Proteins in Hippocampal CA1 Region and Prefrontal Cortical Neurons

**Figure 4:**
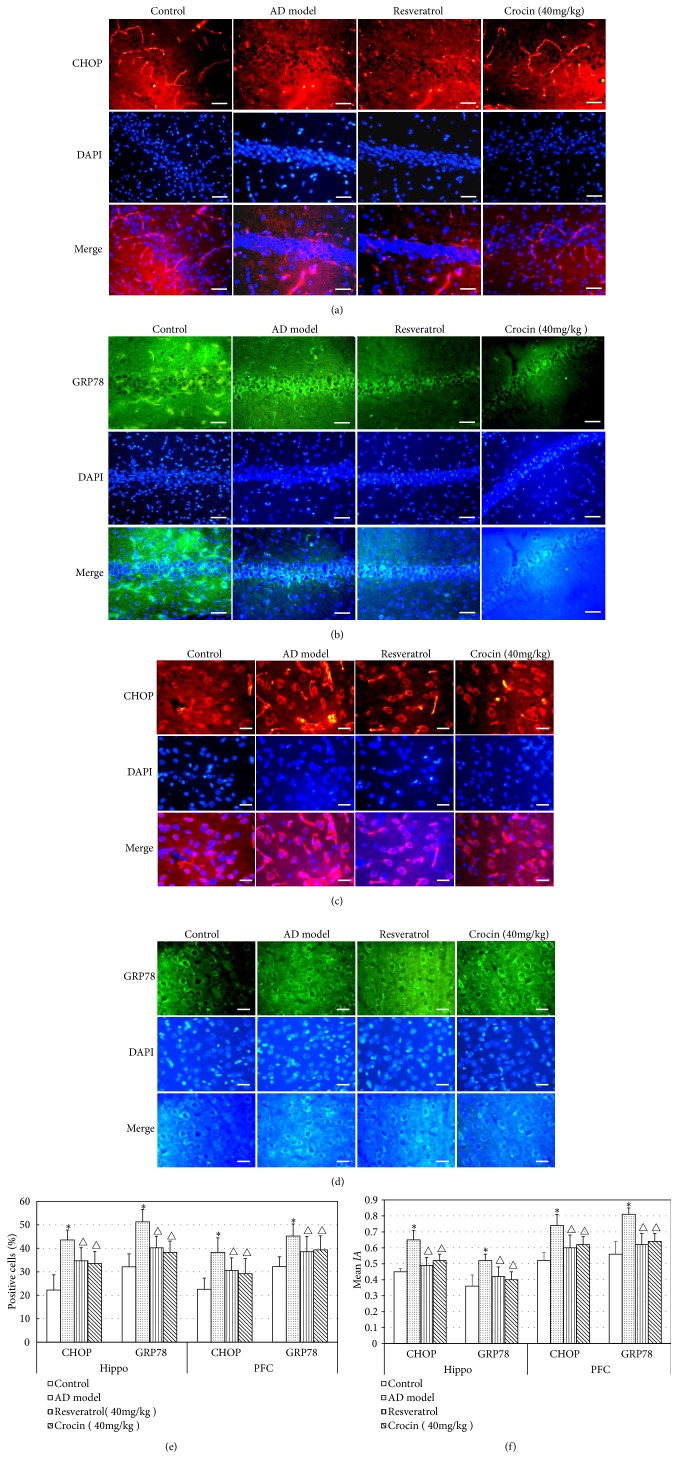
Immunofluorescence staining of CHOP and GRP78 proteins. (a) Immunofluorescence staining of CHOP proteins in CA1 area of Hippo in each group of rats. (b) Immunofluorescence staining of GRP78 proteins in CA1 area of Hippo in each group of rats. (c) Immunofluorescence staining of CHOP proteins in PFC in each group of rats. (d) Immunofluorescence staining of GRP78 proteins in PFC in each group of rats. The scare bar=20 *μ*m. (e) CHOP or GRP78 positive cells in Hippo and PFC in each group of rats (n=6). (f) The mean IA of CHOP and GRP78 expression in hippo and PFC in each group of rats (n=6). Data was shown as Mean±SD. *∗*P<0.05 vs control group; ^△^P<0.05 vs AD model group.

Because excessive ERS is the main cause of apoptosis, we detected the expression of endoplasmic reticulum stress-related proteins GRP78 and CHOP in hippocampal CA1 region (Hippo) and prefrontal cortical neurons (PFC) of rats in each group. As shown in Figures 4(a)–4(d), Cy3-labeled CHOP and FITC-labeled GRP78 proteins could be clearly observed in the cytosol of Hippo and PFC. To interpret the result of immunofluorescence staining, we calculated the number of positive staining cells and mean IA in each group (Figures 4(e) and 4(f)). CHOP and GRP78 positive cells in these two regions of AD rats were significantly increased (P < 0.05) comparing with those of control group. After the treatment of crocin and resveratrol, CHOP and GRP78 positive cells and their expression in hippocampal CA1 tissues and PFC of AD rats were significantly decreased comparing with those of nontreatment AD rats (P < 0.05). Moreover, the effect of crocin and resveratrol was similar.

**Figure 5:**
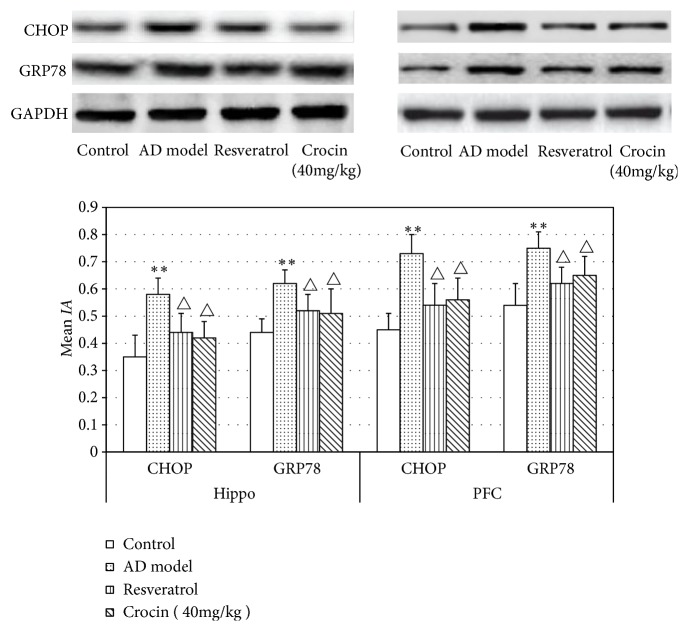
Western blot analysis of CHOP and GRP78 protein expression in Hippo and PFC in each group of rats (n=6). The relative expression of protein was analyzed by comparing with the expression of GAPDH. Data was shown as Mean±SD. *∗*P<0.01 vs control group; ^△^P<0.05 vs AD model group.

We also performed Western blot to validate the expression of ERS-related proteins. As shown in Figure 5, similar results were obtained as that in immunofluorescence staining. In brief, crocin and resveratrol could significantly reverse the increased expression of CHOP and GRP78 in hippocampal tissues and PFC of AD rats (P < 0.05), indicating that crocin can alleviate endoplasmic reticulum stress in Hippo and PFC of AD rats and reduce the expression of GRP78 and CHOP.

## 4. Discussion

In this study, we used the intracerebroventricular administration of *αβ*_25–35_ to induce AD in rats and to investigate the effect of crocin on learning and memory of AD rats by Y-maze test and sucrose preference test. Our results showed that the AD rats attempted significantly more times in both learning and memory stages compared with control rats, leading to significantly lower correct reaction rate. Moreover, the AD rats had less sucrose preference compared with control rats. These results demonstrated that the learning and memory acquisition of AD rats was impaired, and the AD model was successfully established.

Crocin has the functions of lowering blood fat and protecting vascular endothelial cells [16]. Recently it is showed that crocin could alleviate the apoptosis of hippocampal neurons by anti-inflammatory and antioxidative stress abilities [17]. Here we showed that crocin administration significantly improved the learning and memory abilities of AD rats. Both immunofluorescence staining and Western blot showed that the expression of GRP78 and CHOP was significantly increased in hippocampal CA1 region and prefrontal cortical neurons of AD rats, which is consistent with the results of Deng et al. [18]. In contrast, crocin significantly decreased the expression of GRP78 and CHOP. Our data indicate that ERS is activated in the brain neuron of AD rats, which is further rescued by crocin administration.

Bcl2 is an important antiapoptotic protein in cells [19]. A large number of studies [20, 21] have shown that Bcl2 expression is significantly reduced while Bax and Caspase-3, the important proapoptotic factors [22], are increased during apoptosis. In this study, we found that crocin rescued the apoptosis of hippocampal CA1 cells and prefrontal cortical neurons of AD rats, demonstrated by decreased number of apoptosis cells, decreased Bcl-2 expression, and increased Bax and Caspase-3 expression when compared with AD rats. These results are in line with that of Zhang et al. [23], which reported the increased apoptosis of brain neurons in AD rats. In addition, we also demonstrated the protection effect of crocin.

In summary, our study demonstrates that crocin can improve the learning and memory acquisition of AD rats, which is related with its role in alleviating ERS and apoptosis of neuron in the brain of rats. Therefore this study may improve the understanding of AD's pathogenesis and provide basis for treatment of AD.

## Data Availability

The data used to support the findings of this study are included within the article.
